# Burden of post-herpetic neuralgia in a sample of UK residents aged 50 years or older: findings from the zoster quality of life (ZQOL) study

**DOI:** 10.1186/1477-7525-12-92

**Published:** 2014-06-11

**Authors:** Mick Serpell, Adam Gater, Stuart Carroll, Linda Abetz-Webb, Azharul Mannan, Robert Johnson

**Affiliations:** 1 University Department of Anesthesia, Gartnavel General Hospital, Glasgow, UK; 2 Adelphi Values, Cheshire, UK; 3 Sanofi Pasteur MSD, Berkshire, UK; 4 Robert Johnson: University of Bristol, Bristol, UK

## Abstract

**Background:**

Post-herpetic neuralgia (PHN) is the most common complication of herpes zoster (shingles). As a chronic condition, PHN can have a substantial adverse impact on patients’ lives. However, UK-specific data concerning the burden of PHN on individual patients, healthcare systems and wider society, are lacking. As the first UK-wide cross-sectional study of its kind, The Zoster Quality of Life (ZQOL) study was designed to address these concerns.

**Methods:**

Patients (n = 152) with a confirmed diagnosis of PHN (defined as pain persisting ≥ 3 months following rash onset) and aged ≥50 years were recruited from primary and secondary/tertiary care centres throughout the UK. All patients completed validated questionnaires, including the Zoster Brief Pain Inventory (ZBPI), the Medical Outcomes Study Short-Form 36 (SF-36), the EuroQol-5 Dimensions (EQ-5D) and the Treatment Satisfaction with Medication (TSQM) questionnaire. Where available, mean patient population scores on these questionnaires were compared to scores derived from age-matched normative samples to quantify the burden associated with PHN.

**Results:**

Despite numerous consultations with healthcare professionals and receiving multiple medications for the management of their PHN, the majority of patients reported being in pain ‘most of the time’ or ‘all of the time’. A total of 59.9% (n = 91) of all PHN patients reported pain in the preceding 24 hours to assessment at levels (ZBPI worst pain ≥ 5) typically considered to have a significant impact on Health Related Quality of Life (HRQoL). Accordingly, scores for SF-36 and EQ-5D indicated significant deficits in HRQoL among PHN patients compared to age-matched norms (p < 0.05) and patients reported being dissatisfied with the perceived efficacy of therapies received for the management of PHN. Increased pain severity was observed among older participants and higher levels of pain severity were associated with greater HRQoL deficits.

**Conclusions:**

The inadequate relief provided by PHN therapies available in the UK is associated with a significant burden among PHN patients in terms of pain severity and deficits in HRQoL which may persist for years. Therefore, alternative means such as prevention of shingles and PHN, are essential for reducing the impact on individual patients, healthcare systems and society as a whole.

## Background

Herpes zoster (HZ) or “shingles” is a viral condition resulting from reactivation of latent varicella-zoster virus (VZV) which is responsible for childhood ‘chickenpox’. Based on available data (1991–2000), it is estimated that there are 225,000 new cases of HZ in England and Wales alone [1]. Incidence of HZ increases with age, with the majority of cases occurring in patients over 50 years of age [2,3] and the risk of developing HZ being 50% or more in those aged 80 years and over [4]. HZ is characterised by a painful, unilateral, dermatomal rash. This rash most commonly presents on the trunk, but may present across any dermatome; this includes the trigeminal dermatome which extends across the eye and may result in HZ with ocular involvement (i.e. Herpes Zoster Ophthalmicus, HZO). Typically, HZ is an acute experience with the distinctive rash and associated pain resolving within one month of presentation [2]. However, despite treatment and resolution of the HZ rash, approximately 10-20% of all HZ patients [4] continue to experience pain for 90 days or more following HZ rash onset [5], with risks being as high as 60-70% in those aged 60 years and over. This is referred to as post-herpetic neuralgia (PHN) and is the most common complication of HZ [5,6].

PHN is a neuropathic syndrome which manifests as ongoing pain along the cutaneous nerve/s located in the area of prior HZ rash and typically involves one or more of the following types of pain: spontaneous aching or burning; paroxysmal shooting; and allodynia and hyperalgesia [7]. While in the majority of cases this pain will resolve within a year of initial rash presentation, for some patients the pain may persist for five years or more [8]. As expected, given the relationship to HZ, the incidence of PHN increases markedly with age [9,10]. However, incidence is also linked to the severity of the pain experienced during the HZ episode. Those patients experiencing the highest levels of acute pain during HZ presentation are most at risk of developing PHN [11]. PHN is also more prevalent amongst female than male HZ sufferers [12,13].

Past cross-sectional, epidemiological studies have demonstrated that the pain and resulting discomfort associated with PHN have a substantial, and negative, impact on patients’ Health-related Quality of Life (HRQoL) along with their ability to engage in daily activities [7,11,14,15]. As a chronic condition, care provision for PHN patients in terms of visits to primary care (general practitioner centres) and outpatient secondary/tertiary care centres (specialist pain clinics and ophthalmologists), inpatient visits (hospitalisations) and prescriptions constitutes a substantial cost to healthcare systems [13,16-21]. Furthermore, PHN is also associated with important indirect costs, primarily in terms of loss of productivity for patients and caregivers [20,22].

To date, only limited real-world evaluation of the burden of HZ and PHN specific to UK patients has been conducted. Furthermore, the research that has been conducted is limited by relatively small sample sizes and a lack of geographic representation [22]. In particular, information pertaining specifically to the burden of PHN in UK patients is notably absent. To address this, the Zoster Quality of Life (ZQOL) study was designed to assess the clinical characteristics, patient-reported burden and wider societal burden associated with HZ and PHN within the UK. As a chronic condition, the patient-reported impact, clinical management and economic costs associated with PHN are quite distinct from HZ and warrant particular attention. PHN-specific information derived from the ZQOL study is therefore outlined herein.

Therefore, the objectives of the current study were to generate evidence regarding the following:

1. Disease presentation and disease history in UK PHN patients;

2. Burden of PHN for UK patients aged 50 years and over in terms of subjective experience of pain and HRQoL impact;

3. Economic burden associated with PHN in terms of healthcare resource (e.g. prescribed medical interventions, time spent with healthcare professionals etc.);

4. PHN patient satisfaction with prescribed medical interventions.

## Methods

### Study overview

The ZQOL study adopted a cross-sectional observational design involving data collection from HZ/PHN patients and their treating doctors. No medical product or device in addition to standard care was administered to study patients, nor did participation in the study necessitate any change from standard of care.

### Recruitment

A total of 150 PHN patients were targeted for recruitment to the ZQOL to ensure sufficient power for the comparison of Patient Reported Outcome (PRO) data collected from study participants to published aged-matched normative values for the respective instruments. This served as the primary statistical analyses for the study. Adequacy of the study sample size for this purpose was verified via 100,000 Monte Carlo simulations, using the Hochberg (1988) Step-up Procedure to control for family-wise error rate in multiple comparisons [23].

The management of HZ and PHN is largely based in primary care (i.e. general practice), but some patients (particularly those with long-lasting PHN) may be referred to secondary/tertiary care centres (e.g. specialist pain clinics) [24]. Whilst past studies have failed to identify significant differences between patients treated in primary or secondary/tertiary centres [14,25], patients were recruited from a combination of both types of centre throughout England, Scotland, Wales and Northern Ireland, in order to avoid any potential bias in patient selection.

Consecutive patients presenting at the site as part of routine clinical practice were considered for participation in the study. Frequency of presentation of PHN cases was expected to be greater at secondary care centres and therefore fewer secondary care sites than primary care sites were targeted for inclusion in the study. Patient eligibility for participation in the study was ascertained by doctor completion of a study-specific case report form (CRF). There exists no universally agreed definition for PHN, but dermatomal pain or allodynia persisting, recurring or arising 90 days after the onset of the HZ rash is a commonly used definition and considered more conservative than alternative definitions based on persisting or recurrent pain for 30 days. In addition to implementation of this criterion, only patients aged 50 years or over were eligible for participation in this study. This is consistent with data suggesting that the incidence and severity of HZ and PHN increases significantly within this age range [2].

To ensure ecological validity of the study, extensive exclusion criteria (as would typically be adopted in a clinical trial) were not employed. However, to protect the integrity of study findings, PHN patients with health impairments that may make it difficult for them to complete the required battery of PRO instruments (e.g. deficits in cognitive functioning or visual impairments) were excluded from participation. Similarly patients who had taken part in a clinical trial related to HZ/PHN, pain and/or immunomodulation therapy in the past 6 months or who were previously experienced neuropathic pain in the dermatomal region of their HZ rash prior to the onset of HZ were also excluded so to not bias study findings. Of note, PRO instruments were only provided in English and non-English speakers were excluded from participation in the study.

### Study procedures

Prior to recruitment of study patients, all site staff received formal training in study procedures. This included procedures for administering PRO questionnaires to patients to ensure standardisation and minimisation of missing data (e.g. ensuring that patients do not receive additional help from anyone when completing the questionnaires and that all questionnaires are thoroughly checked for completeness). In addition, as a pre-requisite of site participation, all site staff were required to demonstrate evidence of completion of good clinical practice (GCP) training.

Once informed consent had been obtained and participant eligibility confirmed, patients were asked by study staff to complete a combination of existing validated PRO questionnaires as well as a study-specific socio-demographic questionnaire. All questionnaires were bound in a study booklet to ensure standardisation of presentation and order of completion among all patients.

### Study questionnaires

Data were collected using the following PRO questionnaires.

#### Patient socio-demographic questionnaire

Used to collect patient socio-demographic data (age, gender, ethnicity, living situation, education level and employment status) and data concerning patients’ experiences of PHN.

#### Zoster brief pain inventory (ZBPI)

The ZBPI is designed specifically to evaluate pain intensity due to HZ (which may include allodynia and pruritus) and the extent that this may interfere with respondent’s activities of daily living [26]. The instrument comprises 15 items taken from the Brief Pain Inventory-Short Form (BPI-SF), a well-validated PRO measure applied in a broad spectrum of medical conditions [27] and includes four items assessing ‘Pain severity’ and seven items assessing ‘Pain interference’. All items comprising these subscales are assessed via 11-point numerical rating scales (NRS) ranging from 0 (no pain/does not interfere) to 10 (pain as bad as you can imagine/completely interferes). Prior research has shown the ZBPI to be a reliable and valid assessment of pain severity and impact for use in HZ and PHN patients [26] and the instrument has been utilised in prior cross-sectional and prospective investigations [11,22,28].

#### MOS 36-item short-form health survey (SF-36) standard form

The SF-36 is a standardised generic questionnaire that consists of 36 items evaluating HRQoL [29]. The psychometric validity of the SF-36 is well-established. In particular, the SF-36 has demonstrated validity for use in HZ and PHN populations [14,30]. Items explore the following eight dimensions: physical functioning, role functioning due to physical problems, role functioning due to emotional problems, bodily pain, general health perceptions, vitality, social function and mental health. Two summary scores, the Physical Component Summary (PCS) and Mental Component Summary (MCS), can also be calculated. Scores for the eight scale dimensions and two summary scores were calculated using norm-based scoring (NBS) algorithms which employ a linear T-score transformation with mean = 50 and standard deviation = 10 – making it possible to meaningfully compare scores across domains/summary scores [31].

#### The EuroQoL 5 dimensions questionnaire (EQ-5D)

The EQ-5D is a standardised generic questionnaire comprising 5 items that are used to provide a simple descriptive profile and a single index value for health status (ranging from 0–1): EQ-5D Health State Index (HSI) [32]. In addition, the EQ-5D also includes a patient-completed visual analogue scale (VAS), which records the respondent’s self-rated health (SRH) on a vertical scale where the endpoints are labelled from 0 (‘worst imaginable health state’) to 100 (‘best imaginable health state’).

#### Treatment satisfaction questionnaire for medication (TSQM) version II

The TSQM Version II is an 11-item questionnaire designed to assess patients’ satisfaction with various aspects of their medication, including side effects (3 items + 1 yes/no item), effectiveness (2 items), convenience (3 items) and overall treatment satisfaction (2 items) [33]. Items are assessed using 5-point and 7-point Likert scales ranging from ‘Extremely dissatisfied’ to ‘Extremely satisfied’, but scores on all domains are transformed to a 0–100 scale whereby higher scores indicate greater levels of patient satisfaction. The TSQM has not been used previously in HZ or PHN populations, but has been psychometrically validated in a number of diverse patient populations [33].

To complement and facilitate interpretation of PRO data, additional information was collected for each patient via a doctor-completed CRF. This CRF included information concerning patients’ socio-demographic characteristics (e.g. BMI) and clinical presentation of PHN (e.g. time since formal diagnosis). In addition, information concerning patients’ medication and treatment history (including frequency of consultations, hospitalisations, referrals and additional investigations) was also collected.

### Ethics

The ZQOL study was designed and conducted in accordance with the principles set forth in the Declaration of Helsinki [34]. In accordance with current research governance frameworks in the UK, ethical approval of the study was obtained from the National Research Ethics Service (NRES) before data collection began. Furthermore, as the study involved use of National Health Service (NHS) staff, premises, resources and data, local R&D management approval was obtained from the respective NHS local authorities before research began at each site [35]. Finally, informed consent was obtained from patients prior to the collection of data from themselves or their doctor and all data were handled in accordance with the 1998 Data Protection Act.

### Statistical analyses

All planned statistical analyses were specified a priori in a formally developed Statistical Analysis Plan (SAP). All study data were subject to extensive quality control checks prior to the conduct of analyses. In accordance with developer instructions, if missing data for a SF-36 domain was ≤ 50% then data were imputed using the mean of participants’ responses to the completed items [29]. In the absence of developer instructions, missing data for the ZBPI, SF-36 (where >50%), EQ-5D and TSQM were not imputed and, where item scores were missing, relevant domain scores were not calculated.

To quantify the burden associated with PHN, mean SF-36 and EQ-5D scores from ZQOL study samples were compared to published age-matched normative values for the respective questionnaires as primary statistical analyses for the study [36,37]. The statistical significance of differences was investigated via the conduct of unifactorial tests. Differences of equal to or greater than 0.5 standard deviation units of a baseline or comparator score were characterised as clinically meaningful [38-40].

In addition, a number of sub-analyses were conducted upon patient scores on the composite domains of the ZBPI (pain severity and pain interference), SF-36 (PCS and MCS) and EQ-5D (HSI and SRH) to test a number of pre-specified hypotheses. Specifically, prior research has indicated that reports of PHN pain severity increase with age [13]. The association between patient age and reported pain severity, pain interference and HRQoL impact was investigated via calculation of Spearman Rho correlation coefficients. Previous evidence research has indicated differential reports of pain severity, pain interference and HRQoL impact by gender across a range of medical conditions (including HZ and PHN ) [25,41]. To test this hypothesis, mean composite scores among male and female patients were compared using unifactorial tests. Prior research has also indicated that pain interference and HRQoL impact are greatest among patients reporting the greatest levels of pain [15]. To test this hypothesis, ZBPI pain interference, SF-36 and EQ-5D composite scores were compared among groups of patients stratified according to empirically confirmed categorisations of scores on the ‘Worst pain’ item of the ZBPI: none (0); mild (1–4); moderate (5–6); severe (7–10) [42]. ZBPI ratings of ‘worst pain’ were considered as past research has indicated that ratings of ‘worst pain’ are the more reliable than ratings of ‘average’ or ‘current’ pain [26].

Finally, In order to explore potential predictors of pain and HRQOL (as assessed by composite scores for the aforementioned PRO questionnaires), a series of ordinary least squares (OLS) regression analyses were conducted. Independent predictor variables included: socio-demographic variables (age, gender, ethnicity, BMI); time with formal diagnosis of PHN; diagnosis of Herpes Zoster Ophthalmicus (HZO); antiviral prescription within 72 hours of rash presentation; level of analgesics used as defined according to the World Health Organisation (WHO) analgesic ladder (level 1: non-opioid vs. level 2/3: weak/strong opioids) [43]; hospitalisations (binary – Yes/No); co-morbidities; and ZBPI ‘Worst pain’ item scores. Assumptions of the OLS models (specifically normal distribution of errors, collinearity of independent variables and absence of extreme outliers) were verified prior to the conduct of analyses.

Regression analyses were performed using the statistical package R. All other analyses were conducted using STATA software version 10.1

## Results

### Recruitment and participant socio-demographic characteristics

A total of 152 PHN patients were recruited to the ZQOL study from a combination of primary care and secondary care sites between April 2010 and May 2011. A total of 82 patients were recruited from 25 primary care sites with an average of 3.28 patients recruited from each site (range 1–12). A total of 70 patients were recruited from 8 secondary care sites with average 8.75 patients recruited from each site (range 1–20).

Socio-demographic and clinical characteristics for the total study sample are presented in Table 1.

**Table 1:** PHN ZQOL study patients: socio-demographic data

**Characteristic**	**PHN patients (n = 152)**
**Age years** – mean (Range)	71.5 (50 – 96)
**Gender n (%)**	
male	57 (37.5%)
female n (%)	95 (62.5%)
**Body Mass Index (BMI)** – mean (Range)	27.4 (18 – 44)
**Ethnicity n (%)**	
White/Caucasian	150 (98.7%)
Afro-Caribbean	1 (0.7%)
Mixed race	1 (0.7%)
**Comorbidities n (%)**	
Any comorbid disorder	141 (92.8%)
Cardiovascular	98 (64.5%)
Immune	6 (3.9%)
Metabolic/endocrine	39 (25.7%)
Neoplasms	23 (15.1%)
Psychological/psychiatric	46 (30.3%)
Respiratory	20 (13.2%)
Rheumatoid/neurological	88 (57.9%)
**Highest level of education n (%)**	
Secondary school or less	69 (45.4%)
O level or equivalent	21 (13.8%)
A level or equivalent	10 (6.6%)
Vocational	30 (19.7%)
Undergraduate degree	10 (6.6%)
Post graduate degree	9 (5.9%)
Other	3 (2.0%)
**Work status n (%)**	
Full or part time employment	21 (13.8%)
Retired	120 (78.9%)
Unable to work due to post-herpetic neuralgia	3 (2.0%)
Unable to work for other medical reasons	5 (3.3%)
Other	3 (2.0%)
**Living situation n (%)**	
Living alone	53 (34.9%)
Living with partner	90 (59.2%)
Living with partner and children	2 (1.3%)
Living with children	2 (1.3%)
Living with friends	1 (0.7%)
Living in a communal residence	0 (0%)
Living with family	4 (2.6%)
**Years since formal diagnosis of PHN – mean (SD)**	3.57 (5.11)
**Current prescribed treatment by type n (%)**	
Antidepressant	90 (59%)
WHO Level 1 analgesic	84 (55%)
WHO Level 2 analgesic	76 (50%)
Local anaesthetic/analgesic	34 (22%)
Topical analgesic	26 (17%)
WHO Level 3 analgesic	14 (9%)
Miscellaneous	37 (24%)

### Disease presentation

**Figure 1:**
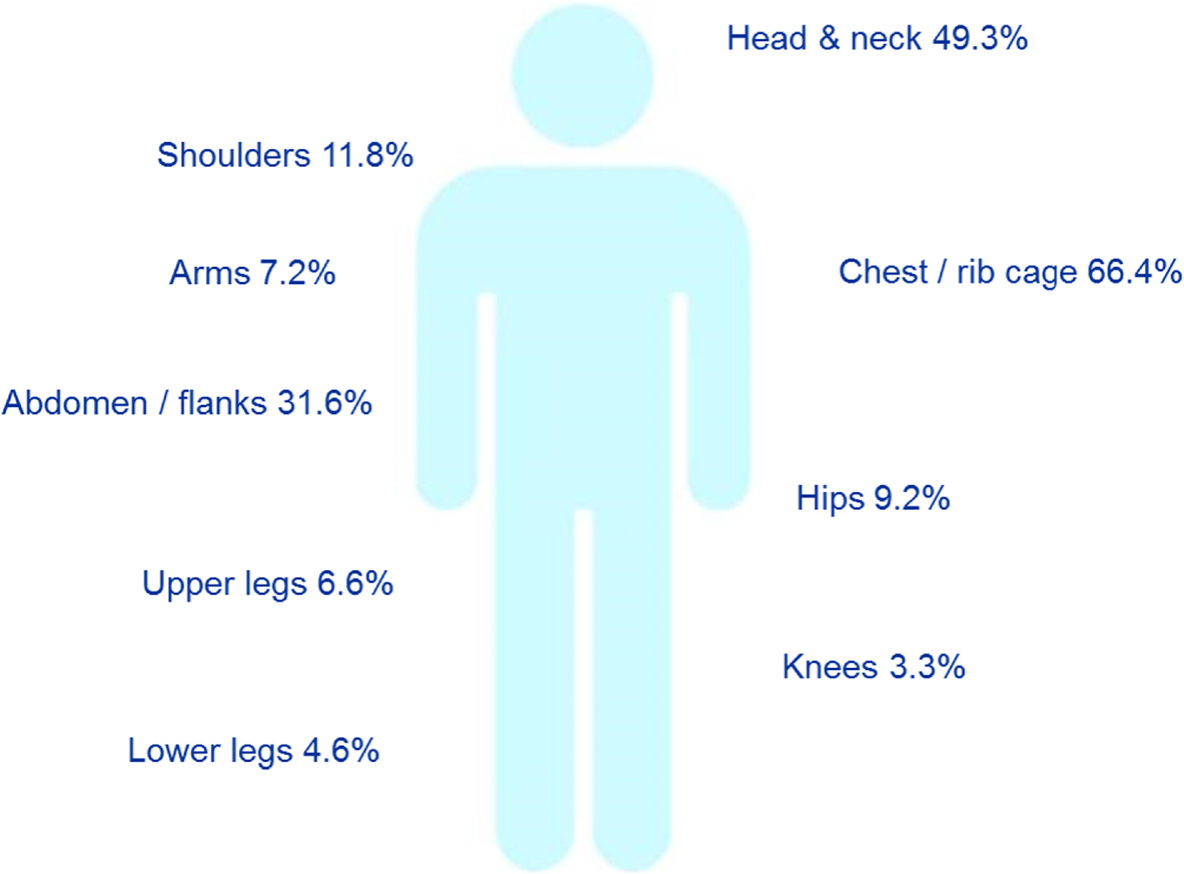
Area of body affected by PHN among ZQOL study patients (n = 152).

PHN patients reported experiencing pain in the area of their HZ rash for an average of 3.5 years (41 months, range 1–473). The majority of ZQOL study participants (59.8%; n = 91) had been suffering from PHN for one year or more. Among ZQOL study patients, the PHN pain most commonly affected more than one site of the body (61.2%; n = 93) and most frequently presented on the chest / rib cage (66.4%; n = 101), head and neck (49.3%; n = 75) and abdomen / flanks (31.6%; n = 48) (see Figure 1).

Reports from patients indicated that this pain was persistent in nature, being present ‘Most of the time’ (36.8%; n = 56) or ‘All the time’ (29.6%; n = 45). The terms most frequently selected by PHN patients to describe their pain were ‘sensitive’ (55.9%; n = 85), ‘tender’ (46.7%; n = 71), ‘burning’ (43.4%; n = 66), ‘aching’ (38.2%; n = 58) and ‘stabbing’ (33.6%; n = 51).

In addition to pain, findings from the ZQOL study reveal that patients with PHN also experience a range of other symptoms. In particular, symptoms of fatigue (61.8%; n = 94), muscle weakness (27.6%; n = 42), change in bowel movements (23.0%; n = 35) and an upset stomach and nausea (23.0%; n = 35) were commonly experienced by study participants.

### Quantification of pain experienced by PHN patients: ZBPI

**Figure 2:**
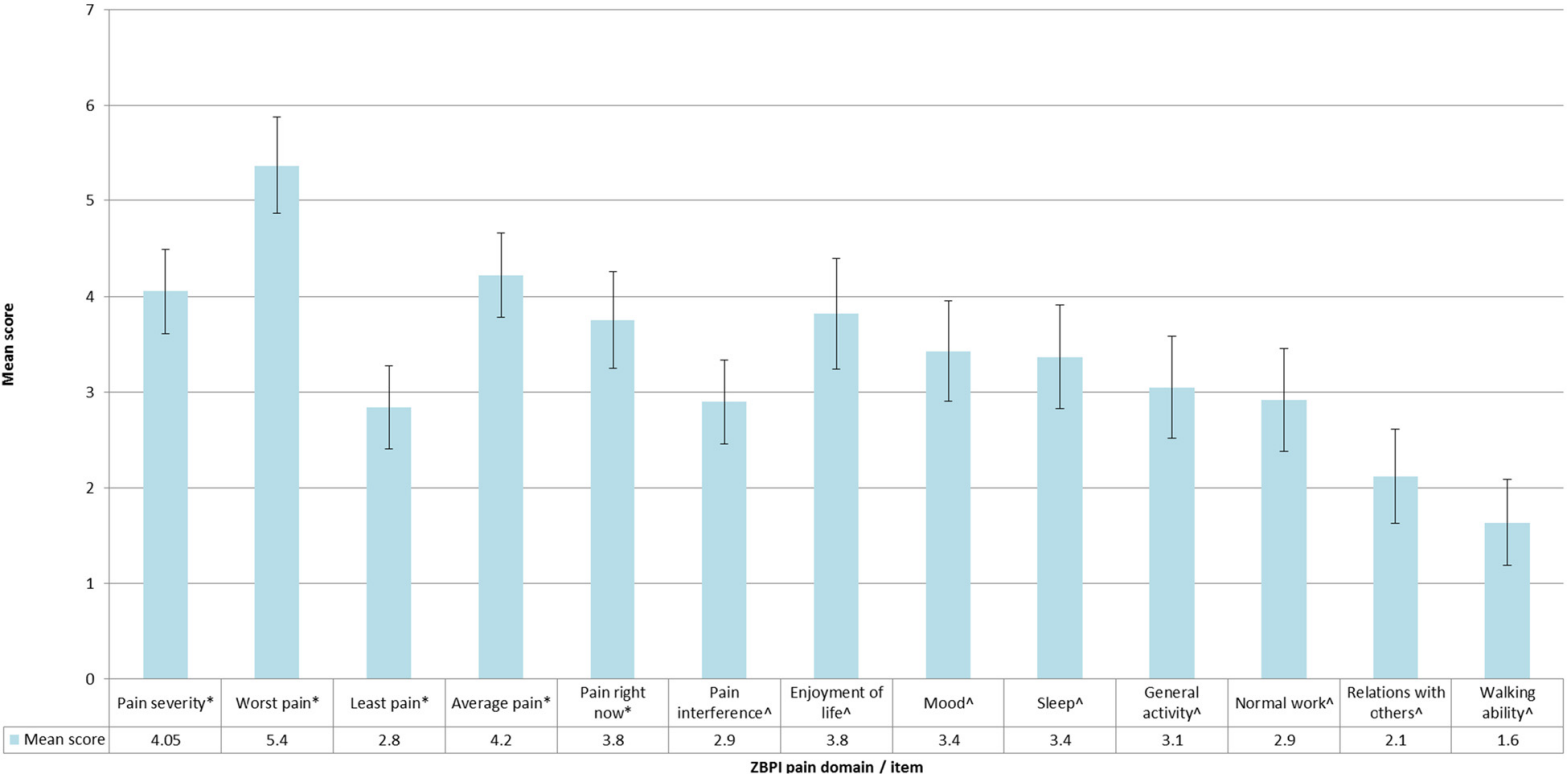
**ZBPI domain and component scores among PHN ZQOL study patients.** Individual items and domains scored 0 (no pain) to 10 (pain as bad as you can imagine) ^ Individual items and domains scored 0 (does not interfere) to 10 (completely interferes).

At the time of the study visit, 80.9% (N = 123) of PHN patients reported experiencing pain in the previous 24 hours and 59.9% (n = 91) of all PHN patients gave ratings of ‘Worst pain’ experienced in the past 24 hours that are indicative of significant HRQoL burden (i.e. worst pain score ≥ 5) [26,44]. Consideration of mean scores for individual items comprising the ZBPI pain interference scale revealed the greatest impact of pain on PHN patients was in terms of ‘Enjoyment of life’, ‘Mood’ and ‘Sleep’ (Figure 2).

Calculation of Spearman rho correlation coefficients indicated a small but statistically significant positive relationship between age and ZBPI pain severity scores. However, no significant association between age and ZBPI pain interference scores were observed (Table 2). Comparisons of ZBPI pain severity and pain interference scores for male and females revealed no significant differences (Table 2). As expected, a significant linear relationship was observed in mean pain interference scores for patients classified according to empirically confirmed cut-offs for pain severity (Table 2).

**Table 2:** PHN pain severity/interference (ZBPI) and HRQOL impact (SF-36 & EQ-5D): ZQOL study subgroup analyses

**Outcome variable**	**Age**		**Gender**			**Pain severity**				
	**Spearman’s rho**	**p**	**Male (n = 57)**	**Female (n = 95)**	**p**	**No pain (n = 15)**	**Mild (n = 42)**	**Moderate (n = 24)**	**Severe (n = 64)**	**p**
Zoster Brief Pain Inventory (ZBPI)										
Pain severity	0.2112	**	4.1	4.0	NS	N/A	N/A	N/A	N/A	N/A
Pain interference	0.1365	NS	2.6	3.1	NS	0.2	1.3	2.8	4.7	***^†^
Short-Form Health Survey 36 (SF-36)										
SF-36 Physical Component Summary (PCS)	−0.2904	***	39.2	37.9	NS	42.1	41.9	37.3	35.5	*
SF-36 Mental Component Summary (MCS)	−0.0527	NS	45.0	43.0	NS	47.8	49.5	46.6	37.7	***^†^
EuroQoL 5-Dimensions (EQ-5D)										
EQ-5D Self-Rated Health (SRH)	−0.1506	NS	63.7	61.0	NS	65.5	69.7	62.0	56.6	*
EQ-5D Health State Index (HSI)	−0.1724	NS	0.54	0.56	NS	0.70	0.73	0.65	0.38	***^†^

NS p ≥ 0.05, * p < 0.05; ** p < 0.01; *** p <0.001. † = clinically meaningful difference.

Prediction of ZBPI composite scores via ordinary least squares (OLS) regression analyses revealed that patient use of level 2/3 analgesics (*r*^2^ = 0.053), patient age (*r*^2^ = 0.043) and time since formal diagnosis of PHN (*r*^2^ = 0.032) were able to explain 13.8% of the variance in ZBPI pain severity composite scores (Table 3). Similar regression analyses revealed that patient ratings of ‘worst pain’ (*r*^2^ = 0.245), use of level 2/3 analgesics (*r*^2^ = 0.052) and the presence of a psychological/psychiatric disorder (*r*^2^ = 0.030) accounted for 38.8% of the variation in pain interference scores.

**Table 3:** PHN pain severity/interference (ZBPI) and HRQOL impact (SF-36 & EQ-5D): ZQOL ordinal least squares (OLS) regression analyses

**Dependent variable**	**Significant predictor (Independent) variables**	**Beta**	**r**^**2**^	**P > [t]**	**95% CI**	**95% CI**	**Model**
					**Lower**	**Upper**	**Adj R**^**2**^
**Zoster Brief Pain Inventory (ZBPI)**							
Pain severity	Analgesic level 2, 3	1.293	0.053	0.004	0.430	2.156	13.8%
	Age	0.056	0.043	0.008	0.015	0.098	
	Length of time with formal diagnosis of PHN	0.008	0.032	0.023	0.001	0.015	
Pain interference	ZBPI Worst pain	2.839	0.245	0.000	2.097	3.580	38.8%
	Analgesic level 2, 3	1.278	0.052	0.001	0.554	2.002	
	Psychological/psychiatric disorder	1.005	0.030	0.009	0.253	1.758	
**Short-Form Health Survey 36 (SF-36)**							
Physical Component Summary (PCS)	Analgesic Level 2, 3	−4.578	0.042	0.009	−7.986	−1.170	12.4%
	Metabolic/endocrine disorder	−4.960	0.041	0.010	−8.719	−1.202	
	Age	−0.203	0.034	0.018	−0.371	−0.035	
Mental Component Summary (MCS)	ZBPI Worst pain	−7.947	0.086	0.000	−11.948	−3.946	23.1%
	Psychological/psychiatric disorder	−8.061	0.082	0.000	−12.226	−3.896	
	Respiratory disorder	6.892	0.031	0.020	1.087	12.698	
	Length of time with formal diagnosis of PHN	−0.034	0.025	0.035	−0.066	−0.002	
	Body Mass Index (BMI)	0.423	0.024	0.039	0.022	0.825	
**EuroQoL 5-Dimensions (EQ-5D)**							
Self-Rated Health (SRH)	ZBPI Worst pain	−8.887	0.044	0.010	−15.577	−2.197	7.3%
	Analgesic level 2, 3	−6.700	0.026	0.045	−13.237	−0.163	
Health State Index (HSI)	ZBPI Worst pain	−0.242	0.133	0.000	−0.337	−0.147	23.3%
	Psychological/psychiatric disorders	−0.141	0.042	0.005	−0.238	−0.043	
	Analgesic level 2, 3	−0.115	0.031	0.016	−0.208	−0.022	

### PHN impact on HRQoL: SF-36

**Figure 3:**
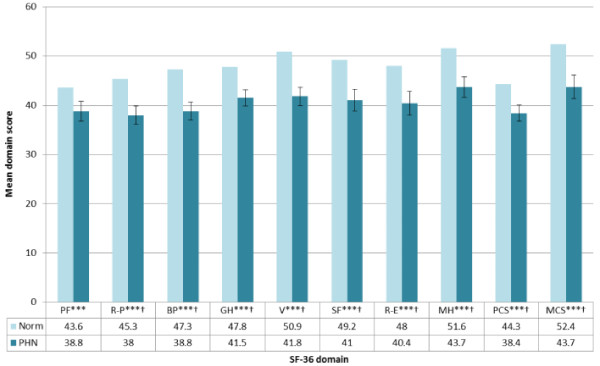
**SF-36 domain and component scores among PHN ZQOL study patients compared to UK population norms.** No *p ≥ 0.05; *p < 0.05; **p < 0.01; ***p < 0.001. † = clinically meaningful. PF = Physical functioning, R-P = Role-physical, BP = Bodily pain, GH = General health, V = Vitality, SF = Social functioning, R-E = Role-emotional, MH = Mental health, PCS = Physical component summary, MCS = Mental component summary.

PHN patients demonstrated significant and clinically meaningful deficits on all SF-36 domain and summary scores (except for physical functioning where only statistically significant deficits were observed) compared to values derived from an age-matched normative population (Figure 3).

Calculation of Spearman rho correlation coefficients revealed a significant inverse relationship between patients’ age and PCS scores (i.e. lower HRQOL with increasing age). However, no significant association between patient age and MCS scores was observed (Table 2). PCS and MCS scores were lower for female PHN patients, compared to male patients. However, differences were not significant. A significant linear relationship was observed between PCS and MCS scores (the latter also clinically meaningful) and patient ratings of ZBPI ‘worst pain’ classified according to empirically confirmed cut-offs for pain severity (Table 2).

OLS regression analyses revealed use of level 2/3 analgesics (*r*^2^ = 0.042) the presence of a metabolic/endocrine disorder (*r*^2^ = 0.041) and patients’ age (*r*^2^ = 0.034) explained 12.4% of the variance in PCS scores (Table 3). Similar regression analyses revealed that patient ratings of ‘worst pain’ (*r*^2^ = 0.086), presence of a psychological/psychiatric disorder (*r*^2^ = 0.082), presence of a respiratory disorder (*r*^2^ = 0.031), time since formal diagnosis of PHN (*r*^2^ = 0.025) and patients’ BMI (*r*^2^ = 0.024) accounted for 23.1% of the variance in MCS scores (Table 3).

### PHN impact on HRQoL: EQ-5D

**Figure 4:**
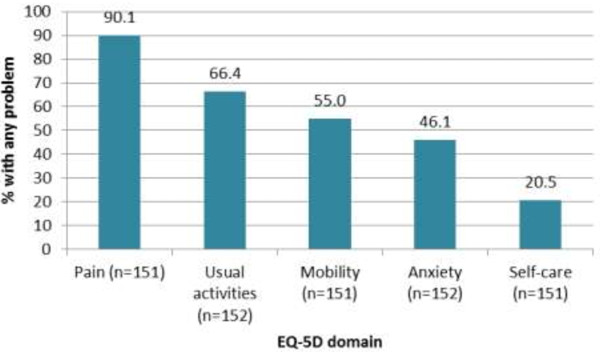
Percent of PHN patients reporting any problem on EuroQol-5 dimensions (EQ-5D) domains.

Consideration of individual EQ-5D scores indicated that ‘Pain’ was the most prevalent problem for patients having been reported by 90.1% of participants (Figure 4). Significant and clinically meaningful deficits were observed for Self-Rated Health (62.0 vs 77.3; p < 0.001) and Health state Index (0.65 vs. 0.78: p < 0.001) scores among PHN patients compared to age matched norms.

Calculation of Spearman rho correlation coefficients revealed no significant association between patients’ age and Self-Rated Health scores. However, a significant inverse relationship was observed between patients’ age and Health State Index scores (Table 2). Comparison of Self-Rated Health and HSI scores for males and females revealed no significant differences. A significant linear relationship between Self-Rated Health and Health State Index scores (the latter of which was also clinically meaningful) was observed for patients classified according to empirically confirmed cut-offs for pain severity (Table 2).

OLS regression analyses indicated that patient-reported levels of ‘Worst pain’ (*r*^2^ = 0.044) and use of level 2/3 analgesics (*r*^2^ = 0.026) were the only significant predictors of Self-Rated Health scores, explaining just 7.3% of the variance in scores (Table 3). By comparison, the presence of a psychological/psychiatric disorder (*r*^2^ = 0.042), patient-reported levels of ‘Worst pain’ (*r*^2^ = 0.133) and patient use of level 2/3 analgesics (*r*^2^ = 0.031) were able to explain 23.3% of the variance in Health State Index scores (Table 3).

### Productivity losses in PHN patients

Only 13.8% of PHN patients (n = 21) in the ZQOL study were currently in full or part-time employment at the time of the study, with the vast majority of participants (78.9%; n = 120) indicating that they were now retired. Despite this fact, considerable productivity losses were evident among ZQOL study participants with reports from participants indicating a mean of 6.4 days (range 0–31) per month when they were unable to take part in their usual activities due to their PHN.

### Impact of PHN on medical resource use

Clinician reports indicated that PHN patients were prescribed multiple medications for the management of their condition. The average number of different medications being taken by ZQOL study participants with PHN was five. Antidepressants (predominantly the tricyclic antidepressant amitriptyline - although not licenced for use as a treatment of PHN in the UK) and anticonvulsants (e.g. pregabalin and gabapentin) were the most common treatment prescribed by doctors for the management of PHN (59.2%; n = 90). Level 1 analgesics (55.3%; n = 84) were the next most commonly prescribed treatment followed by level 2 analgesics (50.0%; n = 76), local anaesthetics/analgesics (22.4%; n = 34) and topical analgesics (17.1%; n = 26). In addition 28.3% (n = 43) of PHN patients reported taking non-prescribed medications for the management of their PHN. The most common non-prescribed medications used by PHN patients were paracetamol (14.9%) and ibuprofen (2.6%). Also of note, consultation of medical records indicated that the majority of PHN patients (59.2%; n = 90) had received an antiviral during the treatment of their initial HZ episode.

Based on patient reports, the average total number of consultations with a healthcare professional related to PHN following diagnosis was 9.5 visits per patient (SD = 19.5). PHN patients for the ZQOL study were recruited via referrals from primary care centres (n = 72) and secondary/tertiary care centres (n = 80). Nearly half (47%; n = 72) of PHN patients, however, had consulted another healthcare professional (most commonly a secondary care pain clinic n = 35) prior to seeing their current healthcare professional. Additional investigations had been requested in 12.5% of patients (n = 19). Investigations requested included full blood counts (n = 16) and tests of renal function (n = 7) and inflammation (n = 6). Only two patients reported being hospitalised due to PHN.

### Treatment satisfaction among PHN patients (TSQM)

**Figure 5:**
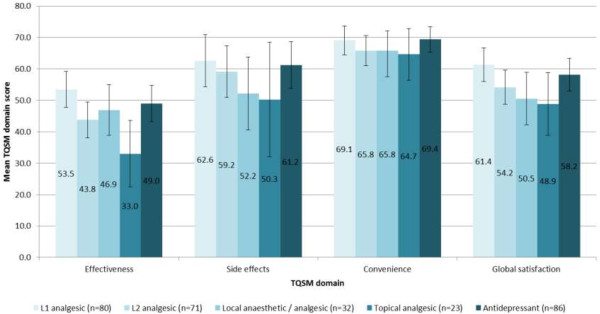
Treatment satisfaction questionnaire for medication (TSQM) domain scores among PHN study patients, categorized according to treatment prescribed for the management of PHN.

PHN patients were classified into five groups according to use of medications for the management of PHN: Level 1 analgesics (e.g. paracetamol, non-steroidal anti-inflammatory drug, Aspirin); Level 2 analgesics (e.g. weak opioids); Local anaesthetic/analgesic; Topical analgesic; Antidepressants/anticonvulsants. Across all groups, TSQM scores indicated that patients were least satisfied with the perceived effectiveness of treatment and most satisfied with the convenience of treatment. No significant or clinically meaningful differences were observed between patients receiving different treatment for the management of their PHN (Figure 5).

## Discussion

While the patient-reported burden of PHN has been explored in past real-world studies, information on the burden of PHN as experienced by UK patients is lacking. The ZQOL study is the first study to evaluate the burden of PHN from the perspective of UK patients, providing data that are important for informing decisions by individual healthcare professionals, local health authorities and national institutions regarding the management of PHN in the UK.

### Understanding the presentation of PHN, experience of pain and HRQOL burden in UK PHN patients

Age and gender distributions of the ZQOL study sample are consistent with prior epidemiological investigations of the incidence of PHN in the UK and previous studies in the US, Canada and Europe [7,11,14,15]. Notably, when compared to the HZ ZQOL study population, PHN patients were (on average) older and more likely to be female [45]. These findings are consistent with prior research identifying age and gender as predictive factors of PHN [46,47].

While PHN has a single well-defined cause, the underlying pathophysiology and presentation may vary between patients and within patients over the course of the disease [48]. Amongst ZQOL participants, PHN was characterised by ‘persistent’ pain most commonly presenting on the chest and ribs (66.4% of cases). Consistent with prior research, the majority of patients (53.3%; n = 81) reported experiencing pain in response to something touching their skin (i.e. tactile allodynia) [49]. Other symptoms (including fatigue, muscle weakness, change in bowel movements and nausea) were also frequently reported and in contrast to prior research which suggests that such symptoms occur in <20% of patients [22].

Past research has demonstrated that patients with facial HZ or Herpes Zoster Ophthalmicus (HZO) are at greatest risk of developing PHN [47]. It is interesting to note, therefore, that a greater proportion of PHN cases were located on the head and neck (49.3%) among ZQOL study participants compared to HZ (30.1%) study participants [45]. Furthermore, while PHN may emerge in a single dermatome, the majority of ZQOL study participants (61.2%; n = 93) felt pain in more than one site of the body with pain often extending to nearby dermatomes. The pervasive nature of PHN is evident by the fact that the majority of patients in the ZQOL study (59.8%) had been suffering from PHN for one year or more (average 3.5 years). This is in contrast to evidence suggesting that PHN resolves within one year in most cases [8] and has considerable implications for patient health and costs associated with medical care.

Results from the ZQOL study indicated that more than half of study participants with PHN (59.9%) reported pain at levels typically considered indicative of significant HRQOL burden [26]. Mean pain scores in this population were similar to those reported in prior investigations in Europe, the US and Canada [7,11,14,15]. Consistent with existing evidence, a significant association between participant age and reported pain severity was observed [13,15]. However, in contrast to prior research that has noted higher reported levels of pain severity in female patients compared to male patients [25,41], no significant gender differences in ZBPI scores were observed among ZQOL study participants.

In the literature published to date, the degree of burden experienced by PHN patients has rarely been interpreted with reference to standardised scores of HRQoL obtained from normative populations [14]. Findings from the ZQOL study indicate that PHN patients demonstrate statistically significant and clinically relevant deficits on all facets of HRQOL (physical and mental/affective components) compared to age-matched UK norms. Of note, in contrast to the findings observed among HZ patients, significant deficits in the SF-36 domain of physical functioning (encompassing difficulties with mobility, self-care and physical activities) compared to age-matched norms were evident among PHN patients [45]. A significant association was observed between the PHN patient’s age and HRQoL deficits on SF-36 domains related to physical functioning only. It is likely that this could be a reflection of general age-related morbidity to which PHN may be a contributory factor. Consistent with the differences observed among normative populations [50], SF-36 and EQ-5D scores were generally lowest among female PHN patients, however differences compared to male PHN patients were non-significant.

### Economic burden of PHN

As a chronic condition, PHN is associated with significant direct and indirect costs, as supported by findings from prior retrospective reviews of medical records and databases [13,16-21]. Direct costs associated with treatment of PHN in the UK (including medication costs, doctor visits and hospital admissions), for example, have previously been estimated at approximately £340 per episode (2009) [13]. Existing data concerning the economic burden of PHN are limited, however, because they are based on retrospective review of data for patients presenting in primary care only. While many patients with PHN are treated in primary care, some patients (particularly those with long-lasting PHN) may be referred to secondary/tertiary care centres (e.g. specialist pain clinics) [24]. Furthermore, such retrospective database reviews only reference direct costs and do not provide an indication of indirect costs which can often be equally as important in chronic diseases. Therefore the true costs and economic burden of PHN may in fact be much higher.

Consultations with healthcare professionals have previously been identified as the primary cost driver in PHN. ZQOL study participants reported having seen a healthcare professional an average of 9.5 times regarding their PHN which supports this notion. Medication costs are another significant driver, again supported by the fact that many participants in the ZQOL study were receiving multiple medications for the management of their PHN. Findings from the study, however, indicated that additional investigations and hospitalisations associated with PHN were a rare occurrence, supporting past research indicating that these factors are only marginal contributors to direct costs associated with PHN [13].

The majority of patients in the ZQOL study were retired and therefore the indirect costs of PHN, specifically in relation to paid employment and work productivity, were minimal. Of the small number of patients who were working, however, almost all reported some impact on their ability to work. As the average age of retirement increases in the UK, it would be reasonable to expect that the impact of PHN on work productivity and absenteeism may become more pronounced in the future. Consistent with deficits reported by PHN patients in relation to physical functioning and mobility, considerable impairments in the ability to complete activities of daily living were also widely reported. Many participants in the ZQOL study were living with someone else and, as a result of these impairments, it is reasonable to expect that a significant proportion of patients may need regular assistance from informal caregivers. The impact of providing care to a patient both during the acute presentation of HZ and during any long-term complications (e.g. PHN), however, remains largely unexplored but may be another contributor to indirect costs associated with the diseases.

### Treatment evaluation

Current therapeutic management of PHN centres upon alleviation of pain associated with PHN. Clinician reports from the ZQOL study supports findings from prior research, which indicates that the treatment of PHN in clinical practice typically requires more than one neuropathic pain medication. A previous investigation by Oster et al. (2005) suggested that patients were generally dissatisfied with their treatment for PHN [15] but to date there has been no attempt to quantify patient satisfaction with HZ and PHN medications using standardised assessments of treatment satisfaction.

Findings from the ZQOL study indicate that despite the availability of various options for pain relief, PHN remains difficult to manage [51,52], with many patients still experiencing significant and clinically relevant levels of pain. As a consequence, patients with PHN report dissatisfaction with the efficacy of current pain treatments. Furthermore, while PHN patients indicate relative satisfaction with the convenience of dosing schedules for pain relief medication, the reliance upon multiple methods of pain relief is a complicating factor for many elderly patients. It can increase the incidence of the side-effects and potential drug interactions associated with concomitant medication use. As many of these patients may also be receiving treatment for a range of co-morbid conditions, the impact of polypharmacy on treatment satisfaction and other patient outcomes should also be considered.

As life expectancy throughout the developed world continues to rise, increasing attention is being paid to the concept of ‘healthy ageing’. Given the challenges associated with managing PHN, preventative strategies for shingles and associated complications (including PHN) should be considered as a means of enabling patients to remain active in old age and minimising the individual patient and societal burden associated with the condition. Existing guidelines for healthcare professionals promote the use of antivirals for the management of HZ episodes [53]. When administered within 72 hours of rash onset, antivirals have been shown to promote rash healing and reduce pain severity in acute HZ [54-56]. However, a recent Cochrane Review found no evidence for an impact of antiviral therapies on PHN, with their efficacy for prevention of PHN considered modest at best [57]. In line with this, 59.2% of PHN patients in the ZQOL study reported having receiving antivirals for the treatment of their preceding HZ episode. There is therefore a need to consider opportunities for prevention and alternative strategies for pain management to reduce the burden of PHN.

The VZV vaccine, Zostavax® (shingles (herpes zoster) vaccine (live)) has been approved by US Food and Drug Administration (FDA) and European Medicines Agency (EMA) for prevention of HZ (“zoster” or shingles) in patients aged 50 years and above. Clinical evidence regarding the efficacy of the VZV vaccine has indicated that in addition to reducing the burden of illness associated with HZ by 61.1%, the vaccine also reduced the incidence of PHN by 66.5% [58]. Use of such a vaccine, therefore, could reduce the burden of illness associated with PHN by reducing the number of incident cases.

### ZQOL study limitations and opportunities for further research

As the first UK-wide of its kind, the ZQOL study provides valuable insights into the burden of PHN in the UK. Nonetheless, there still remain a number of gaps in the understanding of the burden of PHN and opportunities for further research.

Firstly, inconsistencies with disease definition and recognition present challenges to determining the true burden of PHN among UK patients. For example, there exists debate around the definition of PHN. Furthermore, experiences during the ZQOL study, indicate that variation in record-keeping practices between centres (in terms of systems used and information recorded) and the lack of unique identifiers or codes in electronic-record systems present challenges to the identification and recruitment of PHN participants [59]. Past research has also indicated that as many as 80% of patients with PHN may not have this diagnosis specified within administrative systems [18].There is a need, therefore, for greater recognition and appreciation of PHN so as to further understand the true burden of PHN for patients and society.

Secondly, it should be noted that, despite the recruitment of PHN patients from regional sites throughout the UK, the proportion of non-Caucasian patients in the ZQOL study (1.4%) is lower than UK population estimates and less than reported in prior studies in this area [15]. That non-English speaking PHN patients were excluded from the study may, in part, account for the decreased representation of non-white ethnic groups within the ZQOL study. There is also evidence to suggest that VZV infection is less prevalent among certain ethnic groups (e.g. Black African-Americans) [60,61] and those born and raised in non-temperate climates [62]. Nonetheless, as a multi-cultural society, follow-up work to understand the burden of PHN among ethnic minority patients in the UK would be needed to provide a complete understanding of the burden of PHN to UK patients, the healthcare system and society.

Furthermore, it should be acknowledged that there are disadvantages that are inherent to cross-sectional study designs and that apply to the ZQOL study. For example, by investigating patients who already have PHN at a single point in time, it is difficult to infer cause and effect between the development of PHN and HRQOL deficits. Only an association can be determined. In addition, when comparing to age-matched norms the assumption (in the current study) is that observed differences are due to the presence of PHN. However, it must be appreciated that in such studies it is not practically possible to formally account for underlying differences between the study and normative populations or unmeasured factors which may contribute to these observed differences.

Finally, while information collected using formal PRO questionnaires provides a valid means of ‘quantifying’ the burden of HZ, qualitative research exploring the ‘lived experience’ of PHN from a patient’s perspective would be beneficial for further understanding the burden and unmet needs among PHN patients. However, to the authors’ knowledge no qualitative accounts of the experiences of PHN within the scientific literature have been published to date.

## Conclusions

Data from the ZQOL study provide further evidence that, as a common and chronic complication of HZ, PHN has a significant impact of patients’ lives and may result in significant costs for healthcare providers. Data from this study also highlight current unmet needs among PHN patients and inadequacy of current treatments in the management of PHN, reinforcing the need for effective means of prevention and alternative strategies for pain management.

## Abbreviations

BPI-SF: Brief pain inventory-short form; CRF: Case report form; EMA: European medicines agency; EQ-5D: EuroQol-5 dimensions; FDA: Food and drug administration; GCP: Good clinical practice; HRQoL: Health related quality of life; HSI: Health state index; HZ: Herpes zoster; HZO: Herpes zoster ophthalmicus; MCS: Mental component summary; NBS: Norm-based scoring; NHS: National health service; NRES: National research ethics service; NRS: Numerical rating scales; OLS: Ordinary least squares; PCS: Physical component summary; PHN: Post-herpetic neuralgia; PRO: Patient-reported outcome; SAP: Statistical analysis plan; SF-36: Study short-form 36; SRH: Self-rated health; TSQM: Treatment satisfaction with medication; VAS: Visual analogue scale; VZV: Varicella-zoster virus; WHO: World health organisation; ZBPI: Zoster brief pain inventory; ZQOL: Zoster quality of life.

## Competing interests

AG and LA are employees of Adelphi Values, a health outcomes agency commissioned by Sanofi Pasteur MSD, to conduct, analyse and communicate findings from this research on their behalf. SC and AM are employees of Sanofi Pasteur MSD, a provider of a herpes zoster vaccine approved in the European Union. RJ has received consultancy and lecture fees from Sanofi Pasteur MSD, Merck and Merck Frosst. MS has received research support, consulting fees, or honoraria in the past 3 years from Astellas, Astra Zeneca, Grünenthal, Lilly, NAPP and Pfizer.

## Authors’ contributions

AG and LA led the design and conduct of the study, analysis and interpretation of study findings and drafting of the manuscript. SC facilitated the conduct of the study, analysis and interpretation of study findings and drafting of the manuscript. AM, RJ & MS contributed to study design, interpretation of study findings and review of the manuscript from a clinical perspective. All authors read and approved the final manuscript.
